# Editorial: Insights in microbiotechnology-2021

**DOI:** 10.3389/fmicb.2022.1059702

**Published:** 2022-10-31

**Authors:** Ashwani Kumar, Obulisamy Parthiba Karthikeyan, Sanket J. Joshi

**Affiliations:** ^1^Metagenomics and Secretomics Research Laboratory, Department of Botany, Dr. Harisingh Gour University (A Central University), Sagar, India; ^2^Department of Civil and Environmental Engineering, South Dakota School of Mines and Technology, Rapid City, SD, United States; ^3^Institute of Bioresource and Agriculture, Hong Kong Baptist University, Kowloon, Hong Kong SAR, China; ^4^Oil & Gas Research Center, Central Analytical and Applied Research Unit, Sultan Qaboos University, Muscat, Oman

**Keywords:** metagenomics, microbiome, biofilm, microbial diversity, omics

The field of Microbiotechnology has witnessed a significant transition in the last few decades owing to the vast and speedy development of cutting-edge microbial and Omics techniques (Chandran et al., 2020; Kumar and Dubey, 2020). However, Microbiotechnology gained popularity when industrial-scale demands for microbially produced goods led to the new species discovery, the choice of better-known strains, and eventually the introduction of foreign genes to obtain expressed products, novel functional traits, or to increase the strain's capacity and tolerance for product accumulations (Riley and Guss, 2021). Microbiotechnology covers main areas including Food, Agricultural, Chemical and Fuel, Environmental, Medical, and Materials technology (Vitorino and Bessa, 2017). Numerous Microbiotechnological studies have fundamentally altered our knowledge of microbial diversity, evolutionary biology, and molecular microbial interactions with plants, animals, and other organisms (Prasad et al., 2021). The study of natural microorganisms enabled by genomics may help to better understand the genesis, growth, development, and interactions between the biosphere and the environment (Nayfach et al., 2021; Malla et al., 2022). The development of Microbiotechnology sheds light on the numerous microorganisms that have been investigated and used in the creation of various applications, including plant protection and improvement, environmental cleanup, and the enhancement of plant and human health.

Through this call for paper on *Insights in Microbiotechnology-2021* in the journal of *Front. Microbiol*., we invited reviews, mini-reviews, perspectives, and opinions summarizing the field's current state and future directions. This subject focuses on the most significant scientific discoveries, breakthroughs, problems, advancements, and prospects in the area of Microbiotechnology during the year 2021.

Eight articles were published on this Research Topic authored by 44 experts from all over the globe in the Microbiotechnology section of Frontiers in Microbiology. The main goal of these articles is to cover the latest development in the different areas of microbiotechnology. Out of eight articles, four reviews, one opinion, one mini-review, one perspective, and one research article were published. All the articles published were viewed more than 11,200 times. These articles cover different aspects and the latest development in microbiotechnology. The first article in this issue by Raj et al. focused on the application of microbial biosurfactants in bioremediation of pesticides, an environmental friendly method for long-term sustainability. Here authors addressed the function of several biosurfactants produced by the microbes in enhancing pesticide cleanup as well as various techniques ranging from destructive/non-destructive for their detection. This article also discusses how sophisticated metagenomics technologies may be used to profile the bacteria that break down pesticides and produce biosurfactants in various settings. In an opinion paper, Chen and Wang suggested the use of microalgae-based green bio-manufacturing to make a range of biofuels and fine chemicals by genetic engineering modification, namely the gene/genome editing technology, and to generate a number of metabolites through diverse metabolic pathways. Current materials science and synthetic biology techniques for biofilm management were outlined by Shi et al. in order to better manage bacteria and biofilms, the evaluation suggested a viable research area for the future. Núñez et al. evaluated the most recent development in the control of alginate synthesis in a bacterial model system using *Azotobacter vinelandii* as an example. The possibility of producing alginates utilizing bacterial production methods, such as those based on *Azotobacter vinelandii*, was discussed. Zhou et al. presented the idea of a microbial electron transfer network in a viewpoint that further illustrated “species-to-species” connections and discussed various important issues ranging from cellular alteration to microbiome formation. In another article by Vita et al. the bacterial populations in biofilms developing on particles had an improved ability for oil breakdown. The review paper by Roy et al. explored recent findings in direct and indirect electron transfer phenomena that have been revealed by a variety of proteomic, genomic and genetic approaches. Recent developments and publications on synthetic biology and genetic engineering that explore the direct and indirect electron transfer phenomena have been highlighted. Cabugao et al. reviewed the role of popular analytical and omics techniques in understanding the microbial transformation of natural organic matter and its impacts on global carbon cycle. In this way this Research Topic on Microbiotechnology covers various hot topics and latest development in the field that established the ground of future state of the art research in this domain.

## Author contributions

AK prepared the draft version of this Editorial note and all Guest Associate Editors agreed on the final version of the manuscript. All authors contributed to the article and approved the submitted version.

## Conflict of interest

The authors declare that the research was conducted in the absence of any commercial or financial relationships that could be construed as a potential conflict of interest.

## Publisher's note

All claims expressed in this article are solely those of the authors and do not necessarily represent those of their affiliated organizations, or those of the publisher, the editors and the reviewers. Any product that may be evaluated in this article, or claim that may be made by its manufacturer, is not guaranteed or endorsed by the publisher.
